# Developing the First Data-Informed Adult Attention Deficit Hyperactivity Disorder Service in Qatar

**DOI:** 10.7759/cureus.77305

**Published:** 2025-01-11

**Authors:** Ahmad Srour, Oraib Abdallah, Yassin H Eltorki, Abdulkarim Alsiddiqi, Shuja Reagu, Noriya Al-Khuzaei, Majid Alabdulla

**Affiliations:** 1 Psychiatry Department, Hamad Medical Corporation, Doha, QAT; 2 Pharmacy Department, Hamad Medical Corporation, Doha, QAT; 3 College of Medicine, Qatar University, Doha, QAT

**Keywords:** adult adhd, qatar, referral, service development, specialization

## Abstract

Background and objective

The need for developing services to cater to adults with attention deficit hyperactivity disorder (ADHD) was identified following a data review, which revealed a lack of specialized services at Mental Health Services (MHS), the main provider of mental health services in Qatar. This study aimed to gather pertinent data to inform the development of efficient, comprehensive, and reactive care pathways for adult ADHD patient groups in the context of the establishment of the new specialist adult ADHD clinic.

Methods

We conducted a retrospective review of electronic patient records of all patients with adult ADHD. Demographic, sociodemographic, and clinical characteristics of adults diagnosed with ADHD and seen between March 2022 and March 2023 were collected. Descriptive analysis was performed using SPSS Statistics, version 29 (IBM Corp., Armonk, NY).

Results

During the study period, 261 patients were diagnosed (or "diagnosis upheld") with adult ADHD in the specialist clinic and across MHS. The majority (n=141, 54.0%) had ADHD with the type not specified. Most diagnoses were made by psychiatrists (n=165, 63.2%). The endpoint of care data and sources of referrals varied. About 161 (61.7%) were observed to have at least one comorbid condition. Despite being a brand-new initiative, the specialist clinic was fully booked for new cases.

Conclusions

This study is the first of its kind in Qatar examining a cohort of individuals with established adult ADHD diagnoses. It sets out the demographic and clinical data by comparing it to international findings and makes an effective case for a rigorously conducted population-based prevalence study that will inform service pathway development locally and more widely.

## Introduction

Attention deficit hyperactivity disorder (ADHD) is a common inherited neurodevelopmental disorder characterized by a cluster of symptoms such as inattention, hyperactivity, and impulsiveness emerging before the age of 11 years, according to The Diagnostic and Statistical Manual of Mental Disorders, Fifth Edition (DSM-5); it is associated with a high comorbidity of a wide spectrum of mental health disorders including mood disorders, substance misuse, and autistic spectrum disorders [1]. ADHD tends to improve but can continue into adulthood, as almost two-thirds of children diagnosed with ADHD continue to have full syndrome or are in partial remission after the age of 18 years [2]. While overactivity often lessens, impulsiveness, poor concentration, and risk-taking can worsen [1]. ADHD in adult patients remains underdiagnosed and there is a dearth of service for adults diagnosed with ADHD in many countries; despite some individual efforts to develop specialized clinics for adults with ADHD, the provision remains patchy and waiting times can be long [3,4].

Stigma is one of the silent barriers that delay the diagnosis and management of adult patients suffering from ADHD [5]. Also, adults with ADHD are found to have lower-paid jobs and lower socioeconomic status [1]. Lack of training and awareness related to adult ADHD among mental healthcare services has led to this condition being frequently underdiagnosed. This is mainly due to ADHD symptoms being compounded by common comorbid disorders such as anxiety, personality disorders, and mood disorders. The symptoms of ADHD, such as restlessness, mood lability, and inner tension, are then mistaken for comorbid disorders [6]. Lack of education, time and financial limitations, resource constraints, and misconceptions are other barriers that affect the pathway of caring for ADHD [7]. It is estimated that less than 20% of patients with adults with ADHD are accurately diagnosed and properly treated, making many patients vulnerable to developing comorbid anxiety, mood disorders, and substance use disorders, on top of the ongoing social, academic, and occupational challenges [8]. The impact of underdiagnosing and lack of treatment of adults with ADHD is also affecting society in terms of family life, burden on the healthcare system, education, employment, and crime.

There is strong evidence that adults with ADHD respond well to pharmacological treatment, and medications are recommended when ADHD symptoms remain problematic, affecting at least one domain of everyday life despite environmental modifications, as per the National Institute for Health and Care Excellence (NICE) guidelines 2018 [9]. However, assessing, diagnosing, and managing adults with ADHD require the involvement of an entire multidisciplinary team (MDT) [4]. Establishing a specialized clinic for the assessment, diagnosis, and management of adults with ADHD might help with the better utilization of the resources and enhance the training of clinicians involved in the management. In the Arab world, adult ADHD was found to be quite prevalent and associated with high comorbidities and severe impairment; however, it is still under-researched and undertreated [10]. In Qatar, to our knowledge, there have been no published studies exploring the prevalence, patient characteristics, and service provisions for adult ADHD.

In line with Qatar’s public health strategy for 2017-2022 and, more specifically, national mental health strategy, multiple mental health service needs for the population were identified for development by the health leadership [11]. The development of provision for services for adult ADHD was identified following data review and based on the lack of any specialist services at Mental Health Services (MHS), the main provider of secondary and tertiary mental health services in Qatar. A consultant psychiatrist with specialist expertise in diagnosing and treating adult ADHD was tasked with setting up a specialist clinic. The clinic was set up in March 2022 with support from a bespoke MDT, and referral pathways from other parts of the mental health services were developed. Besides service provision, the specialized clinic aimed to gather data to inform the development of efficient, comprehensive, and reactive care pathways for this patient group, in addition to training and enhancing the awareness and skills of mental health clinicians regarding the assessment and management of adult ADHD.

We present the data collected from this clinic during its first year of operation and other MHS clinics at Hamad Medical Corporation (HMC) and discuss how this could shape the development of the first-of-its-kind service in Qatar and the wider Middle Eastern region.

## Materials and methods

Study design, setting, and sample

This study entailed a retrospective review of electronic patient records of all patients referred to the newly set up specialist adult ADHD clinic at Rawdat AlKheil (RAK) clinic in addition to all ADHD-diagnosed patients across HMC. No sampling method was employed as the entire population of interest was considered, and no exclusions were made. The study focused on describing the demographic, sociodemographic, and clinical characteristics of adults aged 18 years and above diagnosed with ADHD and seen between March 2022 and March 2023. The data collection period is inclusive of the follow-up period, and no patient data after the end date was collected. 

Data collection

A dedicated data collection form was developed to facilitate data collection. This form was designed by the research team members to capture information regarding gender, age, nationality, specifics of the ADHD diagnosis (including the diagnosing authority and timing), referral sources, the conclusion of care, and the presence of concurrent psychiatric or neurological/neurodevelopmental conditions.

Ethical consideration

The study was conducted in adherence to the “Declaration of Helsinki”, Good Clinical Practice (GCP), and the laws and regulations of the Ministry of Public Health (MoPH) in Qatar. No intervention was involved, and personal information collected during the study was kept confidential and stored securely. The study sought and obtained approval from the HMC Institutional Review Board (IRB) under the application number MRC-01-23-244.

Data analysis

SPSS Statistics, version 29 (IBM Corp., Armonk, NY) was used for data entry and analysis. We focused on descriptive data as this was an exploratory study. Mean and standard deviations (SD), in addition to frequency and percentages, were calculated. No inferential analyses were applied, given the nature of the study.

## Results

During the study period, 261 patients were diagnosed (or "diagnosis upheld") with adult ADHD in this specialist clinic, and across MHS; 139 (53.3%) of the patients were male. Around 80 (30.3%) of the sample were of Qatari nationality. The majority (141, 54.0%) had ADHD with the type not specified, followed by 75 (28.7%) with the predominantly inattentive type, and 34 (13.0%) with combined ADHD type. The mean age of the sample was 29.32 years (range: 18-62 years. Seventy-six patients (29.1%) were diagnosed with ADHD since childhood and 181 (69.3%) in adulthood.

The diagnoses were primarily made by psychiatrists (n=165, 63.2%), followed by unknown sources (n=81, 31.0%), psychologists (n=13, 5.0%), and other (n=2, 0.8%) practitioners such as pediatricians. ADHD diagnosis was given to 62 (23.8%) of cases by specialists working outside the state of Qatar, while 67 (25.7%) received their diagnosis within the HMC mental health services (including the specialist ADHD clinic), and 48 (18.4%) had received the diagnosis within private settings in Qatar. The source of referrals for ADHD evaluations and assessments came mostly from MHS (n=77, 29.5%), while it was unknown for 51 (19.5%). The endpoint of care data indicates that 195 (74.7%) cases received care at MHS Adult Psychiatry and 56 (21.5%) at the specialized adult ADHD Clinic at RAK Psychiatry ADHD facility, as shown in Table 1.

**Table 1:** Demographics and patient characteristics (N=261) ADHD: attention deficit hyperactivity disorder; CAMHS: Child and Adolescent Mental Health Service; HGH: Hamad General Hospital; HMC: Hamad Medical Corporation; MHS: Mental Health Services; PHCC: Primary Healthcare Center; RAK: Specialized Adult ADHD Clinic Rawdat at AlKheil

Parameter	N (%)
Age group, years	
18-27	138 (53)
28-37	78 (30)
38-47	32 (12)
48-57	11 (4)
58-65	2 (1)
Gender	
Male	139 (53.3)
Female	122 (46.7)
Nationality	
Qatari	80 (30.7)
Non-Qatari	181 (69.3)
ADHD diagnosis	
ADHD in partial remission	4 (1.5)
ADHD type is not recorded	141 (54)
Combined ADHD type	34 (13)
Predominantly hyperactive-impulsive	7 (2.7)
Predominantly inattentive	75 (28.7)
Time of ADHD diagnosis	
Unknown/unclear	4 (1.5)
In adulthood	181 (69.3)
Since childhood	76 (29.1)
Diagnosed with ADHD by	
General pediatrician	2 (0.8)
Psychiatrist	165 (63.2)
Psychologist	13 (5)
Unknown	81 (31)
ADHD diagnosis source	
Abroad	62 (23.8)
Child Development Center - Rumailah	1 (0.4)
Child and Adolescent Mental Health Service (CAMHS) - HMC	9 (3.4)
CAMHS - Sidra Medicine	12 (4.6)
MHS	67 (25.7)
Primary Healthcare Center (PHCC), general	4 (1.5)
PHCC, psych-integrated clinics	13 (5)
Private sector	48 (18.4)
Unknown	45 (17.2)
Source of referrals to MHS-HMC	
CAMHS - HMC	17 (6.5)
CAMHS - Sidra Medicine	12 (4.6)
Child Development Center - Rumailah	2 (0.8)
Direct and private referrals	38 (14.6)
HGH-ED	12 (4.6)
HGH-OPD	7 (2.7)
MHS, general adult psychiatry	77 (29.5)
PHCC, general	34 (13)
PHCC, psych-integrated clinics	11 (4.2)
Unknown	51 (19.5)
Endpoint of care	
HMC general hospitals	10 (3.8)
MHS adult psychiatry	195 (74.7)
RAK Psychiatry ADHD facility	56 (21.5)

A significant portion of cases (n=161, 61.7%) were observed to have at least one comorbid condition, while 100 (38.3%) had no other psychiatric or neurological comorbidities. Comorbid psychiatric conditions included anxiety and anxiety-related disorders in 44 (16.9%), mixed depression with anxiety in 40 (15.3%), depression in 30 (11.5%), and learning disability in 24 (9.2%) patients. Comorbid neurological/neurodevelopmental conditions were less prevalent, with 226 cases (86.6%) having none. Epilepsy and other seizure disorders were the most common type (n=14, 5.4%), as illustrated in Table 2.

**Table 2:** Patient comorbid conditions (N=261)

Parameter	N (%)
Having comorbid conditions (Either psychiatric/neurological)	
Yes, with at least one comorbid condition	161 (61.7)
No comorbidities at all	100 (38.3)
Comorbid psychiatric conditions	
None	108 (41.4)
Anxiety disorders	44 (16.9)
Mixed depression with anxiety	40 (15.3)
Depression	30 (11.5)
Learning disability disorder	24 (9.2)
Personality disorder	14 (5.4)
Bipolar affective disorder	10 (3.8)
Eating disorders	6 (2.3)
Alcohol/substance use disorder	3 (1.1)
Chronic insomnia	3 (1.1)
Others (cyclothymia, gender identity disorder)	2 (0.8)
Comorbid neurological/neurodevelopmental disorder conditions	
None	226 (86.6)
Epilepsy and other seizure disorders	14 (5.4)
Headaches and migraine	9 (3.4)
Back pain	6 (2.3)
Cerebral palsy	5 (1.9)
Autism spectrum disorder (ASD)	4 (1.5)
Disc disease of the neck and lower back	2 (0.8)
Stroke	2 (0.8)
Fragile X syndrome	2 (0.8)
Multiple sclerosis	1 (0.4)
Neuropathy	1 (0.4)
Scoliosis	1 (0.4)
Glaucoma	1 (0.4)
Vertigo	1 (0.4)
Smith-Magenis syndrome	1 (0.4)

## Discussion

The main observation of this study is that there is a demonstrated need to set up specialist ADHD service pathways in Qatar. Despite being a brand-new initiative, the specialist clinic was fully booked for new cases from the start and assessed a maximum of five to six new cases in each weekly clinic. The clinic has a longer waiting list, which has not been looked at in this study. Regarding the characteristics of patients, this study found gender differences and comorbidity trends that reflect global and regional data. It does show that Qatari nationals are over-represented in the patient group; 30% in the patient group, with Qataris making up only around 10% of the total population. This can be explained by the fact that most of the adult immigrants in Qatar are economic immigrants and adult ADHD sufferers belong to poor vocational and economic settings and may have been selected out.

Regarding ADHD subtypes, we found it often challenging to identify them due to limited documentation by the physician providing treatment. For slightly more than half the patients (54%), the subtype was not identified in any of the visits. This is followed by the inattentive subtype, with 27% of the adult ADHD group compared to 13% with the combined type and only 2.7% having hyperactive type. Such results do align with the established fact that inattention symptoms are more likely to present in adult ADHD compared to hyperactive symptoms [12].

As for the age at the time of first diagnosis and diagnosis provider, we had significant limitations due to shortcomings in electronic documentation; 70% of our patient group was likely diagnosed in adulthood, while 30% had carried the diagnosis before the age of 18 years. ADHD being a neurocognitive disorder normally presents in early childhood; however, the timing of diagnosis does not necessarily reflect this. It is possible that, before the age of 18 years, the symptoms were mitigated by the support of family or teachers in school, but later in life, patients have more difficulties with functioning and hence seek help and get diagnosed [13]. Results from the meta-analysis by Caye et al. showed that the severity of ADHD, treatment for ADHD, the presence of comorbid conduct disorder, and comorbid major depressive disorder were identified as factors predicting the persistence of ADHD from childhood into adulthood [14].

Some other suggested reasons for the absence of a larger number of patients identified as transitioning from CAHMS service to adult ADHD clinic include patients growing out of their ADHD, getting treatment in private settings, or traveling out of the country. This is supported by the fact that 70% of our patients are non-nationals, and they might seek treatment in their own home countries. Another possible reason for the challenge in the initial diagnosis and follow-up treatment is the stigma; despite the burden and the high prevalence of the disease, patients diagnosed with ADHD are still at high risk of facing this stigma; the stigma affects both adults and children/adolescents with ADHD and influences the families and people caring for patients diagnosed with ADHD in addition to the authorities’ attitude towards patients diagnosed with ADHD [15].

Furthermore, we found that 63.2% of the patients were diagnosed initially by psychiatrists, whereas in 31% of the cases, we could not identify the profession of the initial diagnosing healthcare provider. This could be due to the fact that for some patients, the initial diagnosis was made many years ago or in a different healthcare system. It is also worth noting that only 5% of the patients were diagnosed by psychologists and less than 1% by pediatricians. The role of psychologists within HMC seems to aid the diagnosis as they run objective tests [e,g., Vineland for measuring adaptive behavior or Adult ADHD Self-Report Scale (ASRS)]; however, to make a diagnosis, they usually refer the patients back to the psychiatrist. In the US, for example, children diagnosed at or after the age of six years, which is the age when most children enter school, were less likely to receive a diagnosis from a psychiatrist compared to those diagnosed earlier than six years of age; instead, they were more likely to be diagnosed by a psychologist in comparison to younger children with ADHD [16]. It follows that it makes operational sense to employ collaborative strategies that involve psychologists, psychiatrists, and other healthcare practitioners for a thorough evaluation and treatment of ADHD [17]. At the same time, the team's familiarity with the patient significantly impacts the quality of care provided.

The data shows that 61.7% did have comorbid psychiatric or neurologic conditions. When looking at specific conditions, the most noted comorbid conditions were anxiety disorders (16.9%), followed by mixed depression/anxiety (15.3%) and depression (11.5%). The presence of comorbidities like anxiety and depression complicates the clinical presentation of adult ADHD, and our results are consistent with those of Mao and Findling [18]. Patients with learning disability are commonly diagnosed with ADHD in the CAMHS population, but in our study, we identified 9.2% of the included patients to have a learning disability. Research indicates that ADHD is often underdiagnosed in adults and individuals of all ages with learning disabilities [19]. Studies have also shown shared cognitive deficits between ADHD and specific learning disabilities, suggesting an overlap in underlying cognitive mechanisms [20].

In terms of comorbid neurological disorders, 13.4% percent were identified to have one or more neurological conditions. Of them, 5.4% had a seizure disorder or epilepsy. Bechtel et al.'s study concluded that there are significant parallels in behavioral patterns, response to medication, and neural activity between ADHD associated with epilepsy and developmental ADHD. This suggests a shared neurobehavioral basis between the two, making the diagnosis and treatment of ADHD in individuals with epilepsy more complex [21]. This highlights the importance of conducting a comprehensive assessment of the common neurobehavioral mechanisms to effectively and accurately address these conditions. A holistic, multidisciplinary, and systematic diagnostic approach helps ensure that patients are being diagnosed correctly and address those with undiagnosed medical conditions that could mimic ADHD-like symptoms.

The establishment of this service has highlighted the prevalence and unmet clinical needs of this patient cohort. Based on these findings, we suggest that future research in Qatar should aim to include patients who are being treated in the private sector as well as to investigate the treatment and burden of symptoms. This is the first study of its kind in Qatar that investigated the characteristics of patients diagnosed with adult ADHD and the existing referral pathways. It should be noted that the patients included in the study may not be representative of the entire adult ADHD population in Qatar, as we could not include data from the private sector. In addition, incomplete medical records limited the ability to comprehensively assess the service utilization patterns as the study was mainly retrospective.

However, this study sets the baseline data scenario for further exploration of the whole population regarding adult ADHD prevalence and the development of effective care pathways encompassing assessment, diagnosis, and recovery for affected individuals. This study makes the case for expanding upon the already established service and comparing the demographic and clinical details regionally to inform and guide service development and public health funding. 

## Conclusions

This observational study is the first of its kind in Qatar to assess a cohort of individuals with established adult ADHD diagnoses. It sets out demographic and clinical data by comparing it to national and international findings and makes an effective case for a rigorously conducted population-based prevalence study that will inform service pathway development locally and more widely. Future initiatives could focus on enhancing the accessibility of the service to ensure that all patients receive timely and appropriate care.

## References

[1] American Psychiatric Association (2025). American Psychiatric Association: Diagnostic and statistical manual of mental disorders. Diagnostic and statistical manual of mental disorders (5th ed., text rev.

[2] Faraone SV, Biederman J, Mick E (2006). The age-dependent decline of attention deficit hyperactivity disorder: a meta-analysis of follow-up studies. Psychol Med.

[3] Schneider BC, Schöttle D, Hottenrott B, Gallinat J, Moritz S (2023). Assessment of adult ADHD in clinical practice: four letters—40 opinions. J Atten Disord.

[4] (2025). Royal College of Psychiatrists in Scotland. Attention deficit hyperactivity disorder (ADHD) in adults: good practice guidelines (2023). https://www.rcpsych.ac.uk/docs/default-source/improving-care/better-mh-policy/college-reports/cr235-adhd-in-adults---good-practice-guidance.pdf.

[5] Schoeman R, Voges T (2022). Attention-deficit hyperactivity disorder stigma: the silent barrier to care. S Afr J Psychiatr.

[6] Ginsberg Y, Quintero J, Anand E, Casillas M, Upadhyaya HP (2014). Underdiagnosis of attention-deficit/hyperactivity disorder in adult patients: a review of the literature. Prim Care Companion CNS Disord.

[7] French B, Sayal K, Daley D (2019). Barriers and facilitators to understanding of ADHD in primary care: a mixed-method systematic review. Eur Child Adolesc Psychiatry.

[8] Rivas-Vazquez RA, Diaz SG, Visser MM, Rivas-Vazquez AA (2023). Adult ADHD: underdiagnosis of a treatable condition. J Health Serv Psychol.

[9] (2025). National Institute for Health and Care Excellence (NICE). Attention deficit hyperactivity disorder: diagnosis and management. NICE guideline [NG87]. https://www.nice.org.uk/guidance/ng87.

[10] Hayek GE, Saab D, Farhat C, Krayem Z, Karam E (2019). Adult ADHD in the Arab World: a review. Archiv Psychol.

[11] (2025). Qatar Public Health Strategy 2017-2022. https://extranet.who.int/ncdccs/Data/QAT_B3_QPHS%202017-2022.pdf.

[12] Wilens TE, Biederman J, Faraone SV, Martelon M, Westerberg D, Spencer TJ (2009). Presenting ADHD symptoms, subtypes, and comorbid disorders in clinically referred adults with ADHD. J Clin Psychiatry.

[13] Moffitt TE, Houts R, Asherson P (2015). Is adult ADHD a childhood-onset neurodevelopmental disorder? Evidence from a four-decade longitudinal cohort study. Am J Psychiatry.

[14] Caye A, Spadini AV, Karam RG (2016). Predictors of persistence of ADHD into adulthood: a systematic review of the literature and meta-analysis. Eur Child Adolesc Psychiatry.

[15] Mueller AK, Fuermaier AB, Koerts J, Tucha L (2012). Stigma in attention deficit hyperactivity disorder. Atten Defic Hyperact Disord.

[16] Visser SN, Zablotsky B, Holbrook JR, Danielson ML, Bitsko RH (2015). Diagnostic experiences of children with attention-deficit/hyperactivity disorder. Natl Health Stat Report.

[17] Pham AV, Riviere A (2015). Specific learning disorders and ADHD: current issues in diagnosis across clinical and educational settings. Curr Psychiatry Rep.

[18] Mao AR, Findling RL (2014). Comorbidities in adult attention-deficit/hyperactivity disorder: a practical guide to diagnosis in primary care. Postgrad Med.

[19] Buckley S, Dodd P, Burke A, Guerin S, McEvoy J, Hillery J (2006). Diagnosis and management of attention-deficit hyperactivity disorder in children and adults with and without learning disabilities. Psychiatr Bull.

[20] Aly HY, Effat S, Azb HM, Abd Elsamei AM (2015). Executive functions among Egyptian children with attention deficit hyperactivity disorder and reading disabilities. Middle East Curr Psychiatry.

[21] Bechtel N, Kobel M, Penner IK (2012). Attention-deficit/hyperactivity disorder in childhood epilepsy: a neuropsychological and functional imaging study. Epilepsia.

